# Slowly seeing the light: an integrative review on ecological light pollution as a potential threat for mollusks

**DOI:** 10.1007/s11356-020-11824-7

**Published:** 2020-12-19

**Authors:** Ahmed A. A. Hussein, Erik Bloem, István Fodor, El-Sayed Baz, Menerva M. Tadros, Maha F. M. Soliman, Nahla S. El-Shenawy, Joris M. Koene

**Affiliations:** 1 Zoology Department, Faculty of Science, Suez Canal University, Ismailia, 41522 Egypt; 2 Theodor Bilharz Research Institute (TBRI), Giza, Egypt; 3 Department of Ecological Science, Faculty of Science, Vrije University, De Boelelaan 1085, 1081 Amsterdam, Netherlands; 4 NAP Adaptive Neuroethology, Department of Experimental Zoology, Balaton Limnological Institute, Centre for Ecological Research, 8237 Tihany, Hungary

**Keywords:** Artificial light, Biorhythm, Mollusca, Reproduction, Snails, Slugs, Zeitgeber

## Abstract

Seasonal changes in the natural light condition play a pivotal role in the regulation of many biological processes in organisms. Disruption of this natural condition via the growing loss of darkness as a result of anthropogenic light pollution has been linked to species-wide shifts in behavioral and physiological traits. This review starts with a brief overview of the definition of light pollution and the most recent insights into the perception of light. We then go on to review the evidence for some adverse effects of ecological light pollution on different groups of animals and will focus on mollusks. Taken together, the available evidence suggests a critical role for light pollution as a recent, growing threat to the regulation of various biological processes in these animals, with the potential to disrupt ecosystem stability. The latter indicates that ecological light pollution is an environmental threat that needs to be taken seriously and requires further research attention.

## Introduction

Natural light is known to be a crucial regulating cue for the biological world and generally acts as a zeitgeber for biological rhythms (Bradshaw and Holzapfel 2010; Foster and Roenneberg 2008; Ragni and Ribera D’Alcalà 2004). As a natural abiotic factor, it is known to influence many behavioral and physiological processes in animals, e.g., reproduction, energy storage, and (neuronal) activity. One important aspect is the seasonal change in light conditions, meaning that even though natural light is not constant but varies over time, this still provides sufficient information to entrain biological rhythms.

Short-term variation in natural light can, for instance, be due to the presence of clouds that block part of the light coming from the sun or stars, and light intensity may change rapidly with increasing sky turbidity (Cronin et al. 2014). As a result, the natural light intensity of the sun can range from 120,000 lx for direct sunlight at noon to less than 5 lx during misty sunsets or sunrises (Gorman et al. 2005). Despite this variation, such light information still serves as a zeitgeber for many of the behaviors that depend on a circadian or circannual rhythm. Nevertheless, in recent years, it has become clearer that the use of artificial light, as part of increased human activity in environments, can affect or even shift the natural rhythmicity of animals (Gaynor et al. 2018).

Nevertheless, in contrast to urbanization effects caused by chemical pollution (Likens et al. 1996), habitat restructuring (Poff et al. 1997), and invasive species (Ricciardi and Rasmussen 1998), those effects caused by light pollution have only been recognized in the past years (Longcore and Rich 2004; Moore et al. 2006; Nightingale et al. 2006). Insects and larger (vertebrate) animals have received attention on how they are affected by such light pollution. However, the second largest group of animals, the mollusks, have been largely overlooked so far. Nevertheless, such animals do use light as a zeitgeber as well. For example, in the freshwater pond snail, *Lymnaea stagnalis*, circannual changes in environmental light conditions can affect reproduction, energy storage, and neuronal activity. For this species, extended photoperiods are associated with precocious sexual maturation and oviposition (Bohlken and Joosse 1981; Dogterom et al. 1983). However, reduced photoperiods are linked to increased glycogen storage and the initiation of overwintering dormancy (Bailey 1981; Hemminga et al. 1985). Such seasonal changes have also been observed in the synaptic connections between the well-studied RPeD1 neuron of *L. stagnalis* and its follower cells (Copping et al. 1999), as well as in how well these snails can deal with anoxic conditions (Buck et al. 2017). One of the very few studies addressing the effects of light pollution in mollusks concluded that their behavioral changes could potentially disrupt interspecific interactions, and thereby ecosystem functioning (Underwood et al. 2017).

In this review, we aim to specifically focus on the potential effects of light pollution on mollusks to inspire and guide research in this direction. To do so, we first define what the term “light pollution” means exactly. Then, we provide a brief overview of the different roles that natural light can play, using some known examples from the animal kingdom and asking whether there is any evidence for this in mollusks. In each section, we also specifically focus on what is known about light and its perception in mollusks (summarized in Table 1) and in which ways these animals can (potentially) be affected by light pollution. This has led us to a conclusion in which we highlight what we think are the most fruitful areas of research to answer some of the pertinent questions.

**Table 1:** Summary of some reported effects of light pollution on mollusk species

Species	Light condition	Behavior or process	Interaction	Effect	Reference
*Aplysia californica*	Short photoperiods	Egg laying	Temperature	Increase	(Wayne and Block 1992)
*Helix aperta*	Light	Juveniles growth	Temperature	No effect	(Benbellil-Tafoughalt et al. 2009)
*Cornu aspersum ( Helix aspersa)*	Choice between light & dim light	Attraction		Prefer light over dim light	(Perea et al. 2007)
		Overwintering dormancy		Initiation	(Bailey 1981)
	Long photoperiods	Egg laying	Temperature	Increase	(Gomot 1990)
*Helix aspersa var. maxima*	Absence of light	Weight (growth)	Temperature	Increased	(Jess and Marks 1998)
*Limax maximus*	Long photoperiods	Growth rate		Higher	(Sokolove and McCrone 1978)
	Long photoperiods	Goands development		Quicker	
*Limax valentianus*	Long photoperiods	Egg laying	Temperature	Increase	(Hommay et al. 2001; Sokolove and McCrone 1978; Udaka et al. 2008)
	Long photoperiods	Egg production	Temperature	Start sooner	
	Long photoperiods	Egg size	Temperture	Larger	
	Short photoperiod	Gonads		Hevier and quicker development	
	Short photoperiod	Growth rate		Higher	
*Lymnaea acuminate*	Red light	Fecundity, hatchability, and survivability	Fed with chlorophyllin	Reduced	(Kumar et al. 2016)
*Lymnaea stagnalis*	Long photoperiods	Sexual maturation and oviposition		Precocious	(Bohlken and Joosse 1981; Dogterom et al. 1983)
	Long photoperiods	Egg-laying	Clean water stimuli	Increase	(Ter Maat et al. 2012, 2007)
	Long photoperiods	Growth rate	Food availability	Faster	(Ter Maat et al. 2007)
	Short photoperiods	Stored energy	Food availability	More	(Ter Maat et al. 2007)
	Short photoperiods	Glycogen storage		Increase	(Hemminga et al. 1985)
	Shadow	Escape behavior		Stimulate	(Takigami et al. 2014)
	Directed light field	Orientation		Attracted	(van Duivenboden 1982)
*Physa integra*	Light	Attraction		Yes	(Clampitt 1974)
*Physa pomillia*	High intensity	Attraction		Yes	(Badman 1966)

## Artificial light and light pollution

The quantity of artificial light can be used as a rough indication for the size and development of contemporary human societies (Cinzano et al. 2001) because the use of light at night has turned out to be fundamental for modern societies (Hölker et al. 2010a). When thinking about such artificial light at night (often abbreviated as ALAN), one should not only think of city lights at night but also lights from traffic, greenhouses, and agricultural systems, for example. In addition, due to the development of the world economy, industrial facilities such as ports, railway yards, and airports are illuminated all the time, as are lit marketing and advertising columns. All these examples of sources of artificial light could increase environmental stress and alter the natural light-dark cycle of organisms (Barré et al. 2020; Baz et al. 2013; Bruce-White and Shardlow 2011; Dominoni et al. 2020; Health Council of the Netherlands 2000; Hölker et al. 2010a, 2010b; Longcore and Rich 2006, 2004; Navara and Nelson 2007; Outen 1998; Perkin et al. 2011; Verheijen 1985, Verheijen 1960).

**Fig. 1:**
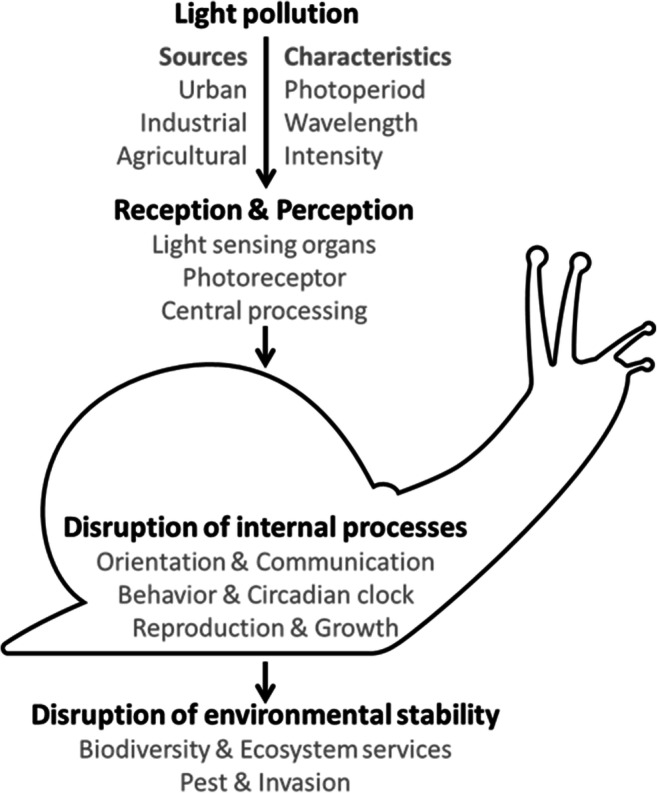
The conceptional framework of ecological light pollution. There are many sources of artificial light at night that can be perceived by animals through different light-sensing organs. The effects of artificial light are mediated by the light photoperiods, wavelength, and/or light intensities of artificial light sources. These characteristics of light may impact separately or combined on one or more internal processes. The combination of two or more of such effects may lead to disruption of environmental stability

The most common definition used for light pollution is the change of natural light patterns in the night environment caused by the introduction of artificial light. Hölker et al. (2010b) showed that the use of artificial lighting is spreading at 6% every year. This can be in the form of direct illumination of the environment surrounding the light sources but can also happen through sky glow resulting from this illumination. Such artificial sky glow can expand the ecological impact of light pollution, as a side effect, to many miles beyond cities. The impressive increase in the use of artificial light at night has put light pollution on the list of threats to ecosystems. Many studies confirm that lighting at night affects wildlife including plants, invertebrates, fish, amphibians, reptiles, birds, and mammals (Barré et al. 2020; Davies et al. 2014; Dominoni et al. 2020; Gaston et al. 2013; Gaston and Bennie 2014; Hölker et al. 2010b; Longcore and Rich 2006; Longcore and Rich 2004), and may have consequences for biological processes (Lewanzik and Voigt 2014). For example, a recent study investigating the mechanisms underlying the near-perfect synchronization of fireflies’ glow also pointed out that some of these species, which use their glow to attract mates, have found themselves competing for attention with human sources of light (Sarfati et al. 2020). Our conceptual framework (Fig. 1) shows how light pollution can affect these processes in terms of changing natural light photoperiod, wavelength, and intensity.

## Quantifying light pollution

One important aspect of light pollution is its quantification. First of all, it is important to differentiate between astronomical light pollution and ecological light pollution, as pointed out by Longcore and Rich (2004). They define astronomical light pollution as the reduction in the visibility of stars originating from artificial light sources at night, a phenomenon also known as sky glow. Next to this, they referred to the effects caused by changes in the natural light levels at night as “ecological light pollution.” In the following, we consider ecological light pollution only.

The quantification of natural light frequently includes measuring the brightening at a given place. There are many ways to express such brightening, but the most widely recognized is the measure of light occurrence per unit of area. Measuring light usually depends on two properties of light: intensity and spectral composition. Nowadays, the most commonly used unit to measure light is lux, which takes the intensity of light into account but neglects the type of light (i.e., the spectral composition represented by wavelengths). Longcore and Rich (2004) suggested that light should be quantified by counting all properties of light. Scientists are therefore advised to quantify brightening in photons per square meter every second accompanied by the wavelengths of that light. This clearly limits the usefulness of lux, especially if one considers that organisms can detect and recognize light at different wavelengths than those visible to humans. Therefore, light with the same intensity but with different wavelengths may have a very different impact on an organism because it depends on that species’ spectral detection range (Dominoni et al. 2020). For example, moths are attracted to high-pressure sodium lights but not to low-pressure sodium lights; these lights have the same lux value but only high-pressure sodium lights produce the ultraviolet wavelength that attracts moths (Rydell 1992).

## Light perception

The previous section illustrated that the physical properties of light can affect organisms in different ways. Therefore, it is important to get a good sense of how organisms use light and darkness as a resource (Gerrish et al. 2009; Kronfeld-Schor and Dayan 2003). The direction, period of exposure, and physical characteristics of light provide organisms with vital information about their surrounding environment, such as locations, the timing of days, different seasons, and years (Neff et al. 2000; Ragni and Ribera D’Alcalà 2004). The usefulness of this information also depends on how efficient organisms are at detecting and recognizing light. Different visual mechanisms have evolved and the level of specialization of visual organs and the accompanying photoreceptors can range from receptors located on the body wall to well-developed eyes (see details in Gehring and Seimiya 2010; Gehring 2014). The origin of vision is assumed to be found in the terrestrial cyanobacterium, *Leptolyngbya* sp., in the form of an eyespot or “stigma” that consists of long, slender trichomes (filaments). This eyespot allowed these bacteria to be positively phototaxic, thus enabling them to move toward a light source. Besides, the eyespot is characterized by containing carotenoid-rich lipid globules that are also found in phototoxic flagellated algae (Albertano et al. 2000).

In general, one of the main reasons for the efficiency or deficiency of vision is related to animal photoreceptor cells and their associated pigments. One important pigment involved in vision is opsin that mediates the conversion of a photon of light into an electrochemical signal as the first step in the visual transduction cascade. The photoreceptor cells in animal eyes are often classified into microvillar cells with rhabdomeric opsin (r-opsin) and ciliary cells with ciliary opsin (c-opsin) according to whether the sensory surface is enlarged by microvilli or by cilia, each type having specialized molecular characteristics (Döring et al. 2020). In response to light, rhabdomeric photoreceptor cells in protostome eyes are known to signal via the Gα_q_-mediated inositol 1,4,5-triphosphate (IP3) cascade opening transient receptor potential (TRP) ion channels in the photoreceptor cell membrane that leads to a depolarization (Fain et al. 2010). In contrast, ciliary photoreceptor cells of vertebrate eyes are known to signal via the Gα_i/t_-mediated cGMP cascade closing cyclic nucleotide-gated (CNG) channels and leading to a hyperpolarization (Nilsson and Arendt 2008; Wensel 2008).

The presence of these two types of receptor cells has been suggested to already be present in the eyes of the bilaterian ancestor for the following main reasons. First, both photoreceptor cells are found in protostome and deuterostome animals and they have distinct molecular characteristics (Arendt 2008; Arendt et al. 2004; Arendt et al. 2002; del Pilar Gomez et al. 2009; Gehring 2014; Panda et al. 2002). Second, c-opsin in protostomes seems to be present only in extraocular photoreceptor cells of certain groups of annelids (Arendt et al. 2004) and arthropods (Beckmann et al. 2015; Velarde et al. 2005) and to be lost in all other protostomes (Döring et al. 2020; Ramirez et al. 2016; Vöcking et al. 2017). This supports the assumption of ancestral extraocular expression of vertebrate c-opsins in brain extraocular photoreceptors (Arendt 2008; Arendt et al. 2004; Shubin et al. 2009) and suggests that the involvement of c-opsins in the visual cells of cerebral eyes evolved later, most likely a chordate-specific phenomenon (Vopalensky et al. 2012).

However, the recent discovery and investigation of a new type of visual opsins, xenopsin, have pointed toward a more complex situation. Xenopsin is present in ciliary photoreceptor cells of a wide range of protostome taxa (summarized in Döring et al. 2020) and although it shares important functional sequence motifs with c-opsins, they do not group in phylogenetic analyses (Ramirez et al. 2016; Rawlinson et al. 2019; Vöcking et al. 2017). Until now, the notion was that xenopsin and c-opsin are mutually exclusive in a given species; however, a new study refuted this view reporting the first organism, the annelid *Malacoceros fuliginosus*, that has both xenopsin and c-opsin (Döring et al. 2020). Furthermore, photoreceptor cells (being potentially polymodal sensory cells) expressing both xenopsin and r-opsin and exhibiting both microvilli and cilia have been found in larva of the mollusk *Leptochiton asellus* and in larva of the annelid *Malacoceros fuliginosus* (Döring et al. 2020; Vöcking et al. 2017)*.*

In the light of these new findings, Döring et al. (2020) have provided a new perspective for comparative eye research: for example, highlighting that xenopsin is an important visual pigment in protostomes and that ciliary eye photoreceptor cells may not be of the same evolutionary origin in protostomes and deuterostomes. As a result, they proposed all conceivable alternative scenarios for the evolution of these opsins and photoreceptor cells in bilaterian animals, which clearly indicated that the exact evolutionary processes remain to be determined and further studies are required for a better understanding.

We will here review some details of the light perception and vision of the Gastropoda (within the phylum Mollusca, the second largest animal group in terms of species number, after insects). The Gastropoda is an extremely diversified class, and its number of species is estimated to lie between 65,000 and 80,000 living snails and slugs. They are often used as bioindicators for the quality of the environment and to detect the effects of different types of pollutants on ecosystems (Bouchet et al. 2005). These species can vary greatly in their behavior, reproduction, habitat, anatomy, and mode of obtaining food. The eyes of a gastropod can be situated at the base of the tentacles, on short stalks, or at the end of their retractable tentacles, respectively, Basommatophora (Hygrophila), and Stylommatophora.

Besides ocular receptors in the eyes, gastropods also have non-ocular light receptors and they use both to avoid total darkness and high intensities of illumination (Brown and Brown 1973; Gotow and Nishi 2009; Lyons et al. 2006; Rossetti and Cabanac 2006). The eye is used for phototaxis and for regulating the behavioral patterns on a daily and seasonal basis. It is still unknown to what extent gastropods can use their eyes to distinguish different shapes and forms. However, they have been shown to distinguish between checkerboard patterns in black and white, as well as a gray background, and they can distinguish between horizontal and vertical lines (Chase 2002). This pattern detection can be explained by the presence of different classes of neurons. For example, Stoll and Bijlsma (1973) presumed that there were 2 classes of neurons in the eye of *L. stagnalis*: photoreceptors and optic ganglion cells. A later morphological examination suggested that there might be 3 types: photoreceptors I and II, and optic ganglion cells (Bobkova 1998). This distinction is not based on the size and area of each type of photoreceptor in the retina but rather on microvilli. The photoreceptor axons extend out of the eye to the central nervous system where they connect with the terminal branches of the statocyst hair cells at a synaptic contact (Sakakibara et al. 2005). It is unclear whether these statocyst hair cells, which are generally involved in balance and orientation (Janse et al. 1988), are also sensitive to light themselves or whether light and other visual information are integrated at this point.

The non-ocular light receptors are known to mediate behavioral responses like the shadow reflex, the defense response when there is a sudden drop in illumination such as that caused by a predator (Ramirez et al. 2011). These non-ocular receptors are distributed over the body wall. Moreover, there is some evidence that when the skin is thin enough, and the light intensity high enough, central neurons can directly be triggered by light (Chase 2002). This direct triggering is hypothesized to be mediated by light-sensitive carotenoids and/or other photopigments that may be present in intracellular neuronal organelles (Gotow and Nishi 2009; Ter Maat et al. 2012) but remains to be demonstrated.

Previous investigations have demonstrated that *L. stagnalis* has TRP-channel-mediated ocular photoreceptor cells and CNG channel-mediated non-ocular photoreceptors. The latter is found especially around the mantle and foot. However, CNG and TRP photoreception can also act synergistically. This happens in the marine gastropod *Onchidium verruculatum* that has additional visual and dermal photoreceptors situated in the dorsal eye and eyestalk. Furthermore, *L. stagnalis* has been confirmed to have three dermal photoreceptors, one in which cyclic guanosine monophosphate acts as a second messenger in the dermal photoreceptor, a second type that contains rhodopsin as a photopigment, and a third that uses the photosensitive protein, Arrestin (Takigami et al. 2014).

## The roles and effects of natural and artificial light on organisms

Organisms use light in many different ways, for example as a resource and also for regulating activity patterns like sleep, reproduction, growth, and orientation. Depending on the kind of physiological process or behavior activity, either light or its absence is a prerequisite. The availability of periods of exposure to light and darkness determines the time that is available for each process. As a result, a disruption in the natural availability of light and darkness can have positive or negative consequences for organisms depending on whether either is a limiting factor.

### Light as a resource and activity determinant

The use of light as a resource is found in the sea slug *Elysia chlorotica* whose metabolism relies on photosynthesis by an alga. This slug feeds on the intertidal alga *Vaucheria litorea*. Instead of digesting the entire algal cell, it conserves the chloroplasts in its gut where they fuse with gut cells and continue photosynthesizing (Mujer et al. 1996). Interestingly, some *Elysia chlorotica* slugs have even reported having the capacity to utilize photosynthesis for up to a year after just a couple of feedings. Such use of light as a resource may provide an example where light pollution may be beneficial since more exposure to light would provide the slug with more energy through photosynthesis (Rumpho et al. 2008).

Light also affects activity patterns of many organisms, with some being active during the day while others being nocturnal (Kronfeld-Schor and Dayan 2003), a separation of activity between organisms that is at least in part due to survival (Gutman and Dayan 2005). However, biological and developmental investigations have concentrated on diurnal organisms, a significant proportion of species adjusted to be active at night during low-light or dark conditions (Hölker et al. 2010b; Lewis and Taylor 1965). These nocturnal organisms seem to be more affected by changes in natural light levels at night in terms of activity, behavior, and survival. Because artificial lighting generally reduces darkness to a semi-darkness level that may be similar to sunset or moonlight conditions—i.e., extending light periods and shortening dark periods—this can lead to changes in behavioral patterns (Mills 2008). Some predators’ ability to detect their prey increases with light level, and indeed some studies considered that such change in light conditions can have a great impact on foraging opportunities, predation, and/or competition (Berger and Gotthard 2008; Falkenberg and Clarke 1998) and can thus influence biodiversity and ecosystem services (Carrascal et al. 2012).

Moreover, it may even create new and unexpected impacts caused by oxidative stress and defects in the pathway of repairing and recovering DNA damage (Queval et al. 2007). The latter can be explained by the need for dark periods, during which damage caused by exposure to solar UV radiation can be fixed (Britt 1996; Sinha and Häder 2002).

### Habitat choice and biodiversity

Organisms can adapt to changes in natural light levels and durations in their environment by leaving light-polluted areas to occupy new habitats. This may make the species invasive in such a new habitat and/or may affect population density levels of their own or native species. González et al. (2014) reported that the occupation level of communities of sandy beach beetles increases with the quality of the sky at night and is thus negatively affected by light pollution due to urbanization. These findings are partly in agreement with those for the impact of light pollution on the amphipod *Orchestoidea tuberculate* (Fanini et al. 2016; Giaconni 2006; González et al. 2014). For black-tailed godwits, there is also suggestive evidence that the nest location depends on the amount of surrounding light, with preferred nesting sites far away from road lighting (De Molenaar et al. 2000). Also, investigations on bats in Sweden demonstrated that artificial night lighting is the reason for a change in bat biodiversity; however, common species remained, but rarer species diminished even more in abundance (Rydell 1992). Finally, within the Mollusca, there is also evidence that the presence of light influences habitat choice. Perea et al. (2007) reported, in a study on the species *Cornu aspersum* (formerly *Helix aspersa*) that were placed in containers with light or dim light conditions, that these snails preferred light over dim light. The latter finding is in agreement with an earlier study by Badman (1966) in which he had found that another species, the freshwater snail *Physa pomillia*, was attracted to the high intensity of light.

### Reproduction

Reproduction is another essential process that can be impacted by changes in natural light patterns in various ways. Females of the frog species *Physalaemus pustulosus* choose a mate more quickly when exposed to higher levels of light, probably to avoid the risk of predation (Rand et al. 1997). In addition to mate choice, the frequency of breeding can also be affected—as Longcore and Rich (2004) found out when studying the effect of sky glow resulting from stadium lighting during football games. In their semi-field experiment, frogs were found not to mate during this lighting period, but they resumed mating after the lighting was blocked by covering their enclosure. Furthermore, McLay et al. (2018) found that *Drosophila melanogaster* flies exposed to 10 lx before mating courted longer than flies exposed to darkness at night before mating. They also found that female oviposition patterns differed between the two light treatments and explained this by determining that female flies exposed to dim light at night had a lower level of reactive oxygen species (ROS) in their ovaries than those exposed to 0 lx.

Egg laying behavior is one of the reproductive parameters that is often influenced by light periods and light pollution. For example, Ter Maat et al. (2007) reported that the period of exposure to light has an effect on egg laying in the snail *L. stagnalis*. They found that snails exposed to long-day conditions (16 L:8D) produce 2- to 3-fold more eggs than normal-day snails (12 L:12D). To confirm their findings on the influence of light on egg laying behavior, they followed up this study by adding the so-called clean water stimulus as an additional factor, which has been shown to induce egg laying when snails are transferred from dirty to clean water and/or jar. They found that when this stimulus was given in the dark, egg laying was induced significantly less than in the light. Hence, light seems to help to stimulate the caudal-dorsal cells (CDCs) that are responsible for releasing the egg laying hormone (CDCH). Interestingly, the snails did not need their eyes to exhibit this difference, so while it remains elusive how light reaches the CDCs it seems likely that this happens via non-ocular photoreception (Ter Maat et al. 2012).

In contrast to freshwater snails, Wayne and Block (1992) stated that for the marine slug *Aplysia californica* exposure to different photoperiods only has a minor influence on reproduction, the main controlling factor being temperature*.* They found that animals that were obtained in autumn and kept in warm water laid eggs more frequently than those in cold water, regardless of photoperiod. This is directly opposed to what Gomot et al. (1989) found in their study on the terrestrial snail *C. aspersum*, which may be down to differences in habitat (i.e., marine vs. terrestrial)*.* The latter study concluded that light is a dominant signaler for inducing egg laying behavior, based on their finding that egg laying is influenced by both temperature and light, but that long-day snails produced more eggs than short-day ones. Moreover, egg laying stopped after 6 weeks of exposure to 15 °C at short days while it continued under normal conditions with 15 °C and long days (which is in agreement with Stephens and Stephens (1966)). Therefore, they conclude that egg-laying hormone production is stimulated by light as in *L. stagnalis*.

Besides egg laying itself, other reproductive parameters can also be affected by light. Kumar et al. (2016) found that the fecundity, hatchability, and survivability of *Lymnaea acuminate* were reduced after being fed with chlorophyllin and exposed to red light. Hommay et al. (2001) also reported that egg production started sooner, more eggs were laid, that these eggs were larger, and that their hatching was significantly higher under long photoperiod when compared to a short photoperiod treatment.

One more reproductive parameter that is greatly affected by light is the growth and development of gonads. Sokolove and McCrone (1978) previously found that *Limax valentianus* slugs that were held under a short photoperiod had gonads and oocytes that were heavier than those from slugs held under long photoperiod. This difference was developmental because it was apparent 90 days after hatching, but disappeared after 120 days and the short photoperiod slugs also reached the last stage of spermatogenesis (Hommay et al. 2001; Udaka et al. 2008).

### Orientation and communication

Light is also an essential player in the orientation of animals, for example to hide from enemies or predators, to locate mating partners, to find food, and/or to migrate; any change in the natural light pattern may alter their perception of direction (Baker 1990). The effect of how migration is affected by artificial lighting at night is found in migratory birds. For example, artificial outside lighting was found to disturb the orientation of young birds, especially in cloudy conditions (Abt and Schultz 1995). Moreover, some migrating birds have been found to fly near lights under bad weather conditions and may get disoriented or even trapped in lit areas (Evans Ogden 1996).

Both diurnal and nocturnal animals are affected by artificial light in their habitat. The increased illumination at night may enhance the ability of diurnal animals to orient themselves and may alter certain behaviors such as foraging in birds (Hill 1992) and reptiles (Schwartz and Henderson 1991). Negative effects of artificial light at night are especially experienced by nocturnal animals because they are adapted to navigate better under dark conditions (Park 1940). One famous example comes from the hatchlings of sea turtles that move from their nests on sandy beaches toward the ocean guided by the natural light, but with the increase of artificial lighting surrounding beaches, the hatchlings get disoriented, and may even move opposite to the direction of the shoreline (Salmon et al. 1995).

By using illumination, animals may also be attracted to or repelled by light sources (Health Council of the Netherlands 2000). A lot of species of insects are attracted to illumination, such as moths. Other taxa like lacewings, beetles, bugs, caddis flies, crane flies, midges, hoverflies, wasps, and bush crickets exhibit similar attraction to light (Eisenbeis and Hassel 2000; Frank 1988; Kolligs 2000). This behavior increases the risk of being predated by bats and spiders (Kiefer et al. 1995; Rydell 1992). In contrast, some nocturnal spiders are negatively phototaxic and repulsed by light (Nakamura and Yamashita 1997) while other insects are positively phototaxic (Summers 1997). In other species the attraction or repulsion effect of light may be used in more applied ways, such as using lights to attract fish to ladders—artificial passages that allow them to bypass dams and power plants (Haymes et al. 1984).

For Mollusca, there is some evidence that light attracts snails (van Duivenboden 1982) and plays a role in predator avoidance (Pankey et al. 2010). *L. stagnalis* can escape from predators via the well-known whole-body withdrawal response. According to behavioral and physiological research, exposure to shadow stimulates snails and their predators in the opposite way. Shadow stimulates the predator to attack, whereas it stimulates non-ocular photoreceptors in the snails to send alert signals to the left and right pedal dorsal 11 neurons. These neurons connect to motor neurons 13–16 via chemical synapses and can initiate the escape behavior (Takigami et al. 2014). This species is even attracted to light when the eyes are experimentally removed (van Duivenboden 1982). The latter clearly indicates that non-ocular photoreceptors are involved. Such positive phototaxis is also seen in the freshwater pulmonate *Physa integra*, which moves toward the shore in spring and is guided by light (Clampitt 1974). Clampitt (1974) found that these snails predominantly moved toward the light and this choice seemed independent of a gravitational cue (Clampitt 1974).

Communication via direct visual cues and/or signals is also used by organisms and may be disturbed by artificial lighting at night. The increased lighting at night in the environment of glowing worms interferes with the attraction of mates via bioluminescence (Lloyd 1994). Hence, the presence of artificial lighting at night decreases the chances of glowing female worms to be located and fertilized by males. So far, there is no evidence from the literature suggesting that light pollution affects sexual signals in mollusks. However, this may not seem surprising for gastropods, which often have limited visual abilities (Di Cristo and Koene 2017). However, it may affect cephalopods since these have well-developed eyesight and are attracted to light. So, this remains a research area that deserves more attention in future studies.

### Disturbance of the circadian clock

There is a crucial role that light plays in synchronizing the nervous system to the external 24-h day-night rhythm (e.g., Baz et al. 2013). The timing of daily rhythms is regulated by an endogenous timekeeping system referred to as the circadian clock. In mammals, this is located in the suprachiasmatic nucleus and in mollusks in the eyes. The biological rhythms differ between organisms and are influenced by periodic cycles (such as day/night, season, high/low tide, and lunar cycle) as well as temperature, wind, and feeding, etc. Many biological functions are linked to such daily or annual periodicity and are controlled by the neuroendocrine system via the epiphysis. This gland is located in the brain and responsible for stimulating the hormonal pathway that produces melatonin (De Molenaar et al. 1997). Artificial illumination at low intensity during the night has been shown to alter secretion of melatonin and thereby internal physiological functions in many species, such as birds, fish, and mammals (Bedrosian et al. 2011a, 2011b; Cos et al. 2006; Evans et al. 2007; Navara and Nelson 2007).

Many studies were done to evaluate the effect of altering melatonin production due to exposure to light pollution. In humans, some researchers have reported a negative relationship between disturbance of melatonin production and cancer risk for people structurally working during the night (Megdal et al. 2005; Reiter et al. 2011; Stevens 2009). At very low-intensity levels of illumination, melatonin secretion may be affected. Rats exposed to illumination intensity of 0.2 lx during the night had decreased levels of melatonin production (Dauchy et al. 1997), similar to the effect of 1 lx on hamster (Brainard et al. 1982), and a higher rate of tumor growth and immune system inhibition were observed (Bedrosian et al. 2011b; Dauchy et al. 1997).

Desynchronization of the biological clock can be one of the impacts that light pollution has (Health Council of the Netherlands 2000). The disturbance may lead to insufficient rest or sleep which may consequently influence fitness and/or survival. Black-tailed godwits and bearded tits can no longer adjust their digestive system for feeding on seeds during winter in Africa from being adjusted to insect feeding during summer due to a lack of synchronization (De Molenaar et al. 2000).

There is no published evidence on the effect of light pollution on the circadian clock of mollusks and/or the secretion of melatonin. This may be because light pollution has only recently been recognized as an environmental threat. Nevertheless, at least one study hypothesized that there are negative effects of disturbance of circadian rhythms of snails due to sky glow resulting from artificial light (Lyytimäki et al. 2012).

### Growth

Growth is also impacted by light pollution. For example, laboratory and field studies by Luarte et al. (2016) revealed that locomotor activity, foraging behavior, and growth rate of the amphipod *Orchestoidea tuberculate* were highly affected by light pollution. In the field, they showed that low light (60 lx) reduced amphipod feeding and growth rates. These findings are in agreement with work showing that light pollution reduces consumption rates in rodents (Vasquez 1994) and decreases the development of juvenile and suckling bats (*Myotis emarginatus* and *M. oxygnathus*) (Boldogh et al. 2007). Similar results are also shown for talitrid amphipods’ growth rate (Duarte et al. 2016).

Raap et al. (2016a, 2016b) tested the effect of artificial light at night on the physiological parameters (body mass and oxidative status) during development, using nestlings of a free-living songbird, the great tit (*Parus major*). They measured multiple biomarkers after two nights of exposure to 3 lx 2 h before sunset and 1 h after sunrise of the following morning. They found that light inhibits body mass but no difference in the oxidative profiles of the exposed individuals. However, this investigation provides evidence that artificial light at night may negatively influence the growth of free-living nestlings that may persist throughout adulthood.

In mollusks, there is also the potential for light pollution affecting growth and development. Ter Maat et al. (2007) discovered that a relationship exists between the daily duration of exposure to light and the growth speed and amount of energy stored in the freshwater pulmonate *L. stagnalis*. Furthermore, the amount of stored energy was higher in the medium-day snails than those in the long-day snails. This is as expected because in spring and autumn food availability is lower; therefore, it is advantageous to store energy whereas in summer food availability is high and there is thus no need to store energy. With a decreasing amount of food available, the dry-weight density of the long-day snails decreased.

For another mollusk, the land snail *H. aspersa* var. *maxima* weight was used as an indicator of growth. The weight increased by 36% in the absence of light at 15 °C compared to snails exposed to 16 h light, while at 20 °C and a light period of 16 h the weight improved by 11% compared to those reared in total darkness. However, at 20 °C, snails were larger in weight by 91% than those raised at 15 °C independent of their photoperiod (Jess and Marks 1998). This finding does not agree with the findings from Benbellil-Tafoughalt et al. (2009) who reported that the growth of juveniles of *Helix aperta* was influenced only by temperature and that exposure to different photoperiods had no effect.

For the terrestrial slug species investigated, growth was greatly affected by exposure to different photoperiods, but in different directions. In *Limax valentianus*, slugs were heavier and had a higher growth rate under short photoperiod (12 L:12D) than those held under long photoperiod (16 L:8D) (Hommay et al. 2001; Udaka et al. 2008). In contrast*, L. maximus* gained more weight in long photoperiods (16 L:8D) compared to short photoperiods (8 L:16D). These opposing findings clearly highlight that the underlying mechanisms may differ, even between closely related species.

## Conclusions and future perspective

The growing use of artificial light at night, such as street lights, greenhouses, industrial facilities, and advertising columns, has the potential to increase the exposure of both aquatic and terrestrial organisms to continuous 24-h photoperiods. This increase could be accompanied by light intensities and spectral compositions, but the real impact of the biological and ecological consequences of artificial night lighting is still unknown. However, researchers are starting to uncover that outdoor illumination affects biological rhythms, and there is a clear need for further exploration of the impact of light pollution on biological systems. Gastropoda seems a suitable class of animals for studying the possible impacts on ecosystems because many members of this group are impacted by changes in natural light regimes (Table 1). As one of the main molluscan model species, *Lymnaea stagnalis* seems highly suitable for testing the effects of light on reproduction, growth, survival rate, and development success because of its demonstrated sensitivity to different light conditions. For this species, exposure to longer photoperiods is already known to enhance and/or initiate various biological processes, such as reproduction (Ter Maat et al. 2012; 2007). Importantly, more work needs to be done to establish whether not only extended constant photoperiods but also lower levels of artificial light at night disturb other processes such as movement activity, behavior, feeding, and ability for learning and memory formation. The latter neurobiological processes involve neuropeptides produced by neuroendocrine cells in the relatively simple central nervous systems (CNS) of *Lymnaea stagnalis*. Their simplicity and well-mapped CNS should enable researchers to better understand the mechanisms responsible for the expected impacts of light pollution on different behavioral and biological processes. In addition, the extensive knowledge about the underlying regulatory mechanisms and the availability of genome and transcriptome data for this species will facilitate interpretation (Fodor et al. 2020).

Earlier research has indicated that light can also have effects that interact with other factors, such as temperature and food availability. While in terrestrial gastropods light seems to be the main trigger, such interaction effects are still largely unexplored in aquatic species and deserve attention in the future, especially given that water temperatures are predicted to rise. Also, from prior research, a primary issue emerges: what consequence does light pollution have on gastropod population density? And does this differ when food is abundant and when food is a limiting factor? Ter Maat et al. (2007) showed that the availability of food and the presence of a longer photoperiod together have a positive effect on the development and reproduction of *L. stagnalis*. This would predict a rise in population density, with the potential to trigger a situation where the species becomes a pest if light pollution continues to expand. The gathering of snails around light sources may increase predation risk, just as it does in moths (Frank et al. 2006). Furthermore, the continued exposure to light may condition gastropods to stop their shadow reflex because of the large amount of false triggering. Combining the latter two might then result in decreased population density, but this also remains to be shown and/or experimentally tested. Hence, the need for further investigation of the effects of a 24-h light period on population dynamics of the freshwater snail through reproduction and behavioral responses like the shadow response, movement activity, and learning becomes necessary for better understanding the implications. We aim to conduct such research in our laboratory in future.

Most reviewed studies only decreased or increased the length of the photoperiod, so it is fair to assume that they used an average light intensity that the gastropods experience in their natural habitat or their culturing facility. Hence, the question remains whether light pollution has a strong enough light intensity to cause a similar effect on these snails as an extended photoperiod at normal intensity. This indicates that in certain animals a low intensity of light pollution is sufficient to change their behavior, so it is entirely possible that gastropods are also affected by such levels of sky glow light pollution and thus remains worth testing. Such research should then focus also on quantifying the lowest level of light necessary to evoke a change in behavior, which will also help to establish the safe limit of light exposure at night in terms of intensity, spectrum, and duration.

Finally, further exploration of this topic will increase our empirical knowledge and help in better understanding the possible impacts of light pollution. Identification of the pigments involved in light perception in mollusks (and animals in general) will also contribute to a more complete understanding of the mechanism and molecular networks underlying the perception and processing of light and help to better identify problematic light levels. Eventually, dealing with ecological light pollution would ideally involve cooperation with physical scientists and engineers to help improve the equipment that can help to avoid ecological light pollution at a critical point in time for ecosystems. Such technical developments are then expected to help control, limit, or even stop the negative impact of light pollution.

## Data Availability

Not applicable
